# Assessment of lotilaner (Credelio® CAT) for control of in-home *Ctenocephalides felis* infestations

**DOI:** 10.1186/s13071-025-06888-8

**Published:** 2025-11-14

**Authors:** Cameron Sutherland, Trey Tomlinson, Grace Wilson, Amiah Gray, Kamilyah Miller, Taylor Gin, Erin Lashnits, Yiyao Li, Todd M. Kollasch, Casey L. Locklear, William G. Ryan, Michael Canfield, Brian H. Herrin

**Affiliations:** 1 College of Veterinary Medicine, Kansas State University, 1800 Denison Ave, Manhattan, KS 66506 USA; 2 College of Veterinary Medicine, University of Florida, 2015 SW 16th Ave, Gainesville, FL 32608 USA; 3 College of Veterinary Medicine, North Carolina State University, 1060 William Moore Drive, Raleigh, NC 27607 USA; 4 School of Veterinary Medicine, University of Wisconsin-Madison, 2015 Linden Drive, Madison, WI 53706 USA; 5 Elanco Animal Health, 2500 Innovation Way, Greenfield, IN 46140 USA; 6 Ryan Mitchell Associates LLC, 16 Stoneleigh Park, Westfield, NJ USA; 7 Animal Hospital Regency Park, 7741 Congress Street, New Port Richey, FL 34653 USA

**Keywords:** Credelio^®^, *Ctenocephalides felis*, Feline, Flea, Flea allergy dermatitis, Isoxazoline, Lotilaner

## Abstract

**Background:**

Flea infestations remain a major issue in veterinary medicine. Highly effective flea control for dogs and cats remains the foundation for eliminating infestations from homes and improving skin conditions associated with flea-feeding.

**Methods:**

Homes with pet cats were screened by flea-history questionnaire. Qualifying homes were subselected into “high” (≥ 5 fleas on ≥ 1 cat, and ≥ 5 fleas collected in environmental flea traps over a 16–24 h period), “low” (< 5 fleas on all cats, < 5 in traps), and “no” homes (no evidence of fleas on cats or traps). All cats and dogs in a household were treated with a lotilaner oral tablet (Credelio^®^ CAT and Credelio^®^, respectively) in weeks 0, 4, and 8. On-animal and trap counts were performed for: “high” at weeks 0, 1, 2, 4, 6, 8, and 11–12; “low” at week 0 and at approximately 2-week intervals through week 11–12; and “no” only at week 0. During each visit, one owner completed a pruritus assessment (PVAS) and a veterinary dermatologist assessed dermatologic lesions using the feline allergic dermatitis (SCORFAD) scale.

**Results:**

A total of 46 homes met inclusion criteria and completed the study: 19 “high” (35 cats); 17 “low” (27); and 10 “no” (14). By week 1, relative to pretreatment, there was a 99.3% reduction in flea counts on “high” cats, with 31 of 34 cats (91.2%) flea-free. By week 11–12, flea counts across all study cats and traps were zero. Prior to the first treatment, mean PVAS scores were: “high” 6.6; “low” 5.5; and “no” 1.9. By week 1 there was a significant decrease in mean PVAS score of cats from “high” homes to 2.9 (*P* < 0.0001), and mean week 11–12 scores were 0.5 and 0.8 for “high” and “low” homes, respectively. For SCORFAD, by week 11–12, relative to week 0, there was a significant decline in mean scores of cats from both “high” (8.0 to 1.7) (*P* < 0.0001) and “low” homes (3.3 to 0.9) (*P* < 0.0001).

**Conclusions:**

Lotilaner was 100% efficacious in eliminating flea infestations from animals and their homes. The monthly lotilaner treatments of cats and dogs in flea-infested homes resulted in clinical resolution of pruritus and dermatologic lesions.

**Graphical abstract:**

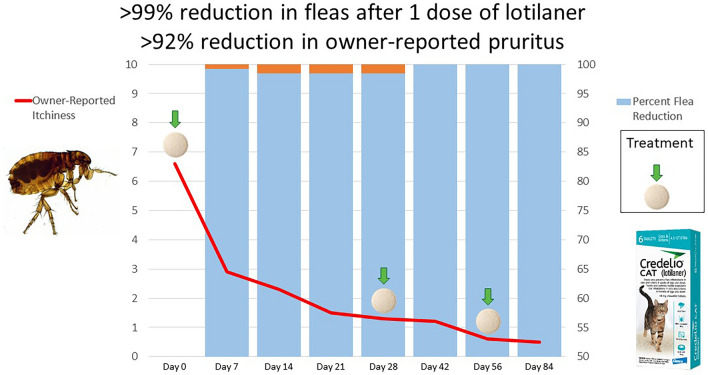

## Background

The cat flea, *Ctenocephalides felis*, is a blood-feeding ectoparasite that can infest a wide variety of mammals, including dogs, cats, and humans [1]. Blood loss arising from heavy flea infestation can result in iron-deficiency anemia [1]. Flea allergy dermatitis (FAD), a hypersensitivity response to antigenic material from flea saliva of the cat flea, is a common cause of pruritic dermatitis in dogs and a major contributor to miliary dermatitis in cats [1–3]. Although recent studies have suggested an association between flea burden and lesions on the ventral abdomen and cervical regions in cats, there is not a recognized pathognomonic reaction pattern for FAD in cats as there is with the dorsal tail base in dogs [4, 5]. Such pruritic skin diseases can impair the quality of life of pets, and of their owners, with potential undermining of the human–animal bond [5–8]. Fleas can also transmit bacterial pathogens (including *Rickettsia felis* and *Bartonella* spp.), and act as intermediate hosts of other parasites (*Dipylidium caninum* and *Acanthocheilonema reconditum*) [9–11].

Historically, cat flea infestations have been challenging to clear from a home due to the continued emergence of adult fleas from the environment and inadequate efficacy of premise sprays. It has now been shown that home infestations can be controlled by treating all pets with a highly effective product that meets success criteria: killing fleas before these can lay eggs (within 24 h of infesting a host) and a speed of flea kill that is then sustained throughout the entire dosing period [12–18]. Fleas emerging from the environment are then killed when finding a treated host, before egg-laying can begin. In addition to eliminating egg-laying, expedient product efficacy is desirable to reduce the allergen load associated with flea burden. These treatments must be continued until the environmental burden of fleas has been eliminated, typically at least 2–3 months [12–18]. Stopping treatment before environmental flea stages are eliminated will result in reinfestation of the animals and subsequently the home. Similarly, without a sustained quick speed of kill throughout the recommended treatment interval, fleas will be able to lay eggs before dying, contaminating the environment with new eggs. Reduced residual speed of kill may be the first sign of a product’s inability to control fleas on cats and dogs, which has historically been documented via post-approval assessment of insecticides through in-home, natural infestations [7, 14]. Thus, in-home evaluation of canine and feline flea control products has become the gold standard for the continual monitoring of product efficacy to confirm a drug class’s ability to continue to kill fleas rapidly throughout the entire dosing period [13, 14, 17, 18].

The isoxazoline drug class has demonstrated efficacy at eliminating home cat flea infestations due to a rapid residual speed of kill [12, 13, 15–18]. This drug class primarily works to inhibit GABA-gated chloride channels of invertebrates, leading to spastic paralysis and death [19]. Lotilaner is an isoxazoline, and the active ingredient in Credelio^®^ CAT (Elanco Animal Health; Greenfield, IN) and Credelio^®^ for dogs and one of the active ingredients in Credelio^®^ Quattro (Elanco Animal Health; Greenfield, IN) for dogs. A previous study showed that home infestations with *C. felis* could be controlled by treating all dogs with lotilaner at monthly intervals for 3 months [15]. In a field study, flea counts of 228 cats, each infested with at least five fleas, were reduced by 98.3, 99.9, and 99.9% on days 30, 60, and 90, respectively, following lotilaner treatment on days 0, 30, and 60 [20]. The current study was designed to build on that finding by investigating whether three consecutive monthly lotilaner treatments of flea-infested cats could eliminate household flea infestations with resulting reductions in pruritus and resolution of skin lesions.

## Methods

### Inclusion criteria

Residential homes in the Tampa area on Florida’s Gulf Coast were recruited in May–June of 2022 through veterinary referrals and Facebook advertisements. Each home was evaluated for inclusion in the study on the basis of a screening questionnaire concerning pet-relevant activities, including time spent indoors and history of flea infestations and control measures. To qualify for enrollment, all household cats and dogs had to spend at least 12 h indoors per day, as reported by the owner, and have no history of flea control measures within 1 month beyond the labeled efficacy duration for the product, and no reported premise treatment within 6 months prior to enrollment. Pet owners had to agree to in-home visits by study staff over the 12-week study. All cats in the home had to be at least 8 weeks old and weigh at least 1 kg, and all dogs at least 8 weeks of age and at least 2 kg. Each enrolled home could have up to 10 dogs and cats in total, but no more than five animals were enrolled for data collection. Any dogs or cats not enrolled but residing within the household were treated with the appropriate lotilaner product on the same schedule as any cat that was enrolled. All animal handling and treatment procedures were reviewed and approved by Kansas State University IACUC #4704 and Kansas State University IRB#: 11,060.

Enrolled homes were categorized as “high” homes, “low” homes, and “no” homes according to the number of fleas present on cats and in environmental traps. For “high” homes, selection criteria included at least five fleas on at least one cat and; at least five fleas in total collected on environmental traps over a 16–24 h period. For “low” homes, the number of fleas on a cat or on environmental traps was lower than five but greater than 0. For “no” homes, no fleas were found on any animals or environmental traps. Any home with dogs or cats that were pregnant, nursing, fractious, or had a history of seizures was excluded from the study. All owners, regardless of study group, signed an informed consent form and questionnaire pertaining to the history of the animals and the home.

### Flea population assessment

The flea population of every cat enrolled in the study for data collection was assessed using a flea combing technique described in a previous study [21]. For pathogen testing at a later date, a subset of fleas was removed from cats with ≥ 10 fleas and the remainder were returned to the animal: for cats with 10–14 fleas, 3 were removed; 15–24 fleas, 6 removed; 25–49 fleas, 9 removed; ≥ 50 fleas, 12 fleas removed.

The environmental flea population in each participating home was assessed using validated, intermittent light traps [22]. Two traps were placed in separate rooms for a 16–24 h period. The trap locations were selected on the basis of owners’ observations of where animals spent most of their time and placed in the same location for each subsequent collection. Fleas collected on the adhesive pads of the traps were counted and morphologically identified.

The on-animal flea counts and flea-trap placements were conducted for “high” homes prior to treatment in week 0, and in weeks 1, 2, 4, 6, 8, and 11–12; for “low” homes in weeks 0, 2, 4, 6, 8, and 11–12; and for “no” homes in week 0 only.

### Evaluation of skin disease

During each collection period, the same owner completed a pruritus visual analog scale (PVAS) on a non-numeric data capture form for each enrolled cat [12]. The PVAS is a validated scale that assesses the owner’s impression of pruritus for dogs [23, 24]. While not validated for cats, previous publications have used such a scale to assess feline pruritus [12, 13, 25]. The pet owners were blinded to the number scale (0 – 10) that was subsequently superimposed over the form to numerically assign a pruritus level.

A board-certified veterinary dermatologist assessed dermatologic lesions on every cat in the home using the scoring feline allergic dermatitis (SCORFAD) scale score for ten different anatomical regions in weeks 0, 4, 8, and 11–12 [26]. These anatomical regions were assessed for excoriations, miliary dermatitis, eosinophilic plaques, and self-induced alopecia on a 0–4 scale.

### Treatments

All cats and dogs in the household were treated with a lotilaner oral tablet, Credelio^®^ CAT and Credelio^®^, respectively (Elanco Animal Health, Greenfield, IN, USA) at weeks 0 (day 0), 4, and 8 according to product label instructions. All animals were offered food by the owner 30 min before treatment. The dosing for each animal was conducted on a dose-banded system according to the product labels, and animals were weighed on a calibrated scale before each dose administration. This resulted in a minimum lotilaner dosage of 20 mg/kg and 6 mg/kg to dogs and cats, respectively. All animals were treated either directly by or under the direct supervision of study staff following product label directions. All animals that received lotilaner treatment were monitored for adverse events. Any adverse event that occurred was recorded and the relationship to treatment evaluated. No other topical or systemic products were used to address dermatologic lesions, inflammation, or secondary bacterial infections associated with flea feeding.

### Data analysis

The outcomes of interest in this study included flea counts on animals, flea counts on traps, PVAS scores, and SCORFAD assessments. The percentage changes in flea burdens from pretreatment counts in week 0 were calculated on the basis of the geometric means, and the changes in PVAS and SCORFAD between pre- and posttreatment assessments were compared using arithmetic means. Geometric means for flea and trap count data were calculated in R by means of the ln (count + 1) transformation using the “psych” package [27]. Spearman’s correlation test was also performed in R and used to assess the relationship between flea counts and PVAS scores, and PVAS and SCORFAD scores within “high” and “low” homes.

## Results

### Homes

From 64 homes that were initially visited, 55 met the inclusion criteria, of which 46 were included in the analysis dataset: 19 “high,” 17 “low,” and 10 “no” homes. Two homes in the “high” category were excluded from the analysis, one because of the introduction of flea-infested kittens too young for treatment and the other because changing home circumstances prevented completion of study procedures. Seven “low” category homes were excluded from the analysis, either because the owners could not adhere to the schedule or because the study cats could not be found. No homes were removed due to the presence of fleas or adverse events. Of the 17 “low” homes, at the enrollment visit 12 had at least 1 cat with 5 or more fleas but fewer than 5 fleas in traps placed in the home. The remaining five “low” homes had fewer than five fleas counted on any cat and collected in traps.

### Animals

Across the 46 qualifying homes for data collection, 76 cats were included in the study calculations: 35 cats in the “high” home category, 27 in the “low”, and 14 in “no” homes. Including the additional household pets that were not enrolled for data collection, 146 cats and 69 dogs were treated with Credelio^®^ CAT and Credelio^®^, respectively. The Credelio^®^ CAT dosages averaged 10.5 mg/kg (range 6.2–22.2 mg/kg) across all cats treated, and the Credelio^®^ dosage averaged 29.0 mg/kg (range 20.2–50.8 mg/kg) across all dogs treated. Treatments were well tolerated by dogs and cats, and there were no treatment-related adverse events.

### Flea counts

The week 0 geometric mean flea count on study cats in “high” homes was significantly greater than on cats in “low” homes (W = 757; *P* < 0.001) (Table 1). By week 1, relative to mean pretreatment counts, there was a 99.3% reduction in fleas counted on “high” home study cats, and 91.2% of these cats (31/34) were flea-free. Between week 0 and week 11–12 there was a complete (100%) and statistically significant (V_H_ = 496, V_L_ = 253; *P* < 0.001) reduction in flea counts in both the “high” and “low” home categories.

**Table 1:** Descriptive statistics summary of on-cat flea counts in study cats

	Week of visit							
	0	1	2	3	4	6	8	11–12
High-flea homes								
Geometric mean	12.7*^‡^	0.1^‡^	0.2^‡^	0.2^‡^	0.2^‡^	0.0^‡^	0.0^‡^	0.0^‡^
Standard deviation	10.1	0.6	0.7	1.0	0.5	0.0	0.2	0.0
Arithmetic mean	15.2	0.2	0.4	0.3	0.2	0.0	0.0	0.0
95% CI	9.1–16.0	0.0–0.3	0.0–0.5	0.0–0.5	0.0–0.3	0.0–0.0	0.0–0.1	0.0–0.0
Range	5–44	0–3	0–2	0–4	0–2	0–0	0–1	0–0
% Reduction compared with week 0^a^	–	99.3	98.2	98.7	98.7	100.0	99.8	100.0
Low-flea homes								
Geometric mean	3.9*^†^		0.0^†^		0.0^†^	0.0^†^	0.0^†^	0.0^†^
Standard deviation	6.7		0.0		0.0	0.0	0.0	0.0
Arithmetic mean	6.4		0.0		0.0	0.0	0.0	0.0
95% CI	1.6–6.7		0.0–0.0		0.0–0.0	0.0–0.0	0.0–0.0	0.0–0.0
Range	0–33		0–0		0–0	0–0	0–0	0–0
% Reduction compared with week 0^a^	–		100.0		100.0	100.0	100.0	100.0

*CI* confidence interval

High-flea homes visited on all shown weeks; Low-flea homes not visited on weeks 1 and 3; no-flea homes not included in table

^a^On the basis of geometric means

^*^Between-group (high–low) difference significant *P* < 0.001; ^‡^Mean flea-count reduction significant (*P* < 0.001) between week 0 and all other weeks

^†^Mean flea-count reduction significant (V = 253; *P* < 0.001) between week 0 and all other weeks

The week 0 geometric mean flea count in environmental flea traps for “high” homes was significantly different from the count in “low” homes (V = 190; *P* < 0.001) (Table 2). By week 1, in “high” homes there was an 82.3% reduction (V = 171; *P* < 0.001) in geometric mean flea-trap counts and by week 3 the reduction was 94.0% (V = 171; *P* < 0.001) (Table 2). Between week 0 and week 11–12 there was a complete (100%) and statistically significant reduction in flea-trap counts in both the “high” (V = 171; *P* < 0.001) and “low” home categories (V = 36*; P* = 0.010).

**Table 2:** Descriptive statistics summary of flea counts from household flea traps

	Week of visit							
	0	1	2	3	4	6	8	11–12
High-flea homes								
Geometric mean	27.4*^‡^	4.8^‡^	3.4*^‡^	1.6^‡^	0.9*^‡^	0.2^‡^	0.1^‡^	0.0^‡^
Standard deviation	85.9	12.1	30.1	20.2	7.5	1.7	0.4	0.0
Arithmetic mean	50.5	10.3	14.4	6.6	2.7	0.5	0.2	0.0
95% CI	9.1–91.9	4.4–16.1	8.6–20.2	0.0–17.2	0.0–6.3	0.0–1.3	0.0–0.4	0.0–0.0
Range	7–387	0–33	0–114	0–89	0–33	0–7	0–1	0–0
% Reduction compared with week 0^a^	–	82.6	87.6	94.0	96.9	99.2	99.6	100.0
Low-flea homes								
Geometric mean	0.6*^†^		0.1*^†^		0.2*^†^	0.0^†^	0.4^†^	0.0^†^
Standard deviation	1.6		0.7		1.4	0.0	0.3	0.0
Arithmetic mean	0.7		0.3		0.6	0.0	0.1	0.0
95% CI	0.2–1.1		0.0–0.6		0.0–0.7	0.0–0 0	0.0–0.2	0.0–0.0
Range	0–3		0–1		0–1	0–0	0–1	0–0
% Reduction compared with week 0^a^	–		79.3		62.9	100.0	93.4	100.0

*CI* confidence interval

^a^On the basis of geometric means

High-flea homes visited on all shown weeks; low-flea homes not visited in weeks 1 and 3; no-flea homes not included in table

^*^Between-group (high–low) difference significant *P* < 0.001; ^‡^Mean flea-count reduction significant between week 0 and all other weeks (*P* < 0.001)

^†^Mean flea-count reduction significant between week 0 and all other weeks (*P* = 0.010)

### Pruritus visual analog scale (PVAS)

Prior to the first treatment, the arithmetic mean PVAS score for cats from “high” homes was 6.6, for cats from “low” homes 5.5, and for cats from “no” homes 1.9 (Table 3). At this point there was no significant difference between the mean PVAS score of cats from “high” and “low” homes (W = 593; *P* = 0.088), but mean scores of cats from both “high” and “low” homes were significantly higher than cats from “no” homes (1.9) (W_H_ = 457, W_L_ = 342; *P* < 0.0001) (Table 3). By week 1, relative to week 0, there was a significant decrease in mean PVAS score of cats from “high” homes (V = 600.5; *P* < 0.0001). By week 11–12, the arithmetic mean PVAS score for cats from “high” homes was not significantly different from the score of cats from “low” homes (W = 291; *P* = 0.067). The week 11–12 mean PVAS scores for “high” and “low” cats were both significantly lower than the mean PVAS score of “no” home cats (i.e., flea-free cats) reported at the start of the study (W_H_ = 59, W_L_ = 90.5; *P* < 0.05), as well as significantly different from their respective week 0 values (V_H_ = 461, V_L_ = 378; *P* < 0.0001).

**Table 3:** Descriptive statistics summary of PVAS of study cats scored by cat owners

	Week of visit							
	0	1	2	3	4	6	8	11–12
High-flea homes								
Mean (SD)	6.6 (2.5)^a‡^	2.9 (1.7)^‡^	2.3 (2.2)^‡^	1.5 (2.2)^‡^	1.3 (1.6)^‡^	1.2 (1.3)^‡^	0.6 (0.8)^‡^	0.5 (0.9)^∞‡^
Median	6.2	2.5	1.8	0.4	0.7	0.4	0.3	0.2
95% CI	5.7–7.5	2.3–3.5	1.6–3.1	0.7–2.3	0.5–1.9	0.7–1.7	0.2–1.0	0.1–0.8
Range	0–9.8	0–5.2	0–9.7	0–9.6	0–6.5	0–3.7	0–2.4	0–4.9
Low-flea homes								
Mean (SD)	5.5 (2.3)^a†^		2.0 (1.7)^†^		1.7 (1.3)^†^	1.5 (1.5)^†^	0.9 (0.9)^†^	0.8 (0.9)^⁑†^
Median	5.0		2.0		2.2	0.7	0.5	0.5
95% CI	4.6–6.4		1.4–2.7		1.2–2.2	0.9–2.1	0.5–1.2	0.4–1.1
Range	0–0.7		0–7.6		0–4.9	0–4.8	0–2.6	0–2.6
No-flea homes								
Mean (SD)	1.9 (1.4)^b∞ ⁑^							
Median	1.7							
95% CI	1.1–2.7							
Range	0–5.1							

*PVAS* pruritus visual analog scale, *SD* standard deviation, *CI* confidence interval

High-flea homes visited in all shown weeks; low-flea homes not visited in weeks 1 and 3; no-flea homes visited only in week 0

^a,b^Mean PVAS scores in week 0 with different letters are significantly different (*P* < 0.0001)

“High” and “low” week 11–12 were significantly different to week 0 “no” homes ^∞^*P*_*H*_ < 0.05; ^⁑^*P*_*L*_ < 0.05

^‡^Mean PVAS reduction significant between week 0 and all other weeks (*P* < 0.001)

^†^Reduction significant between week 0 and all other weeks (*P* < 0.001)

### Scoring feline allergic dermatitis (SCORFAD)

Across the duration of the study, a SCORFAD score was assigned to 35 cats from “high” homes, 26 from “low” homes, and 12 from “no” homes. Before the first treatment, the arithmetic mean SCORFAD score of cats from “high” homes was significantly greater than that of cats from “low” homes (W = 614.5; *P* < 0.05) and “no” homes (W = 353.5; *P* < 0.05), and the mean SCORFAD of cats in “low” homes was significantly higher than those of “no” home cats (W = 226.5; *P* < 0.05) (Table 4). By week 11–12, relative to week 0, there was a significant decline in mean SCORFAD scores of cats from both “high” (V = 351; *P* < 0.0001) and “low” (V = 134, *P* = 0.0007) homes. There was no statistically significant difference in week 11–12 scores between cats in “high” and “low” homes (W = 432; *P* > 0.1). For cats in each of those categories, the mean week 11–12 SCORFAD score was not significantly different from that the enrollment SCORFAD score of cats in “no” homes (W_HN_ = 222.5, W_LN_ = 177.5; *P* > 0.1).

**Table 4:** Descriptive statistics summary of veterinary dermatologist SCORFAD scoring of study cats

	Week of study			
	0	4	8	11–12
High-flea homes				
Mean (SD)	8.0 (8.1)^a‡^	4.3 (5.7)^‡^	2.7 (4.5)^‡^	1.7 (3.0)^‡^
Median	5.5	2.0	1.0	1.0
Range	0–33	0–20	0–24	0–15
95% CI	5.1–10.8	2.3–6.3	0.8–4.7	0.6–2.9
Low-flea homes				
Mean (SD)	3.3 (2.3)^b†^	1.3 (1.4)^†^	1.0 (1.6)^†^	0.9 (0.5)^†^
Median	3.0	1.0	0.0	0.0
Range	0–12	0–5.0	0–6	0–3
95% CI	1.9–4.7	0.7–1.9	0.3–1.7	0.5–1.3
No-flea homes				
Mean (SD)	0.9 (1.4)^c^			
Median	0.0			
Range	0–6			
95% CI	0.0–2.1			

*SD* standard deviation, *CI* confidence interval, *SCORFAD* scoring feline allergic dermatitis

High-flea homes visited in all shown weeks; low-flea homes not visited in weeks 1 and 3; no-flea homes visited only in week 0

^a,b,c^Mean SCORFAD scores in week 0 with different letters are significantly different (*P* < 0.05)

^‡^Mean SCORFAD scores significantly different between week 0 and all other weeks: *P* < 0.0001

^†^Mean SCORFAD scores significantly different between week 0 and all other weeks: *P* < 0.0015

High-flea homes visited on all shown weeks; low-flea homes not visited in weeks 1 and 3

Spearman’s correlation test found no correlation between mean flea counts and PVAS scores prior to the first treatment for either “high” (r(32) = −0.0265; *P* = 0.882) or “low” homes (r(25) = 0.2604; *P* = 0.1896). There was also no correlation detected between cat flea counts and SCORFAD scores in either the “high” home (r(32) = −0.0371; *P* = 0.8378) or “low” home (r(24) = 0.2277; *P* = 0.2632) categories. There was a moderate correlation between the week 0 “high” PVAS and SCORFAD scores r(32) = 0.59, *P* = 0.0002, but that was not seen for the “low” group (r(24) = 0.077; *P* = 0.9702). That correlation seen within the “high” group at week 0 was not found at any other timepoint r(32) < 0.15, *P* > 0.40.

## Discussion

The mean flea counts found in this study are consistent with findings from previous studies on household cats, and lower than those reported from dogs in similar studies, likely attributable to feline grooming behavior [12–18]. Most cats (> 90%) in “high” and “low” homes were flea-free within 1 week of the first treatment, confirming lotilaner’s rapid onset of efficacy. However, fleas were found on treated cats in a few homes past that first week. In week 8, fleas were also found in traps in each of those homes. Thus, the cat infestations would have been due to larvae that hatched and pupae that emerged from the prestudy environmental contamination with flea eggs. The timing of this emergence is based on a variety of factors including temperature, humidity, and pressure-sensory cues that stimulate adult fleas to emerge from the cocoon [22]. Overall, complete clearance of on-animal infestations and environmental burden of fleas in homes in Florida takes approximately 2–3 months, yet in cooler, damper conditions, adult flea emergence can occur over a period as long as 6–9 months (MW Dryden, personal communication). Lotilaner has been shown to kill fleas before egg-laying begins, and the subsequent complete elimination of fleas (from cats and traps) indicates that the fleas found on those cats died before adding to the environmental contamination [28].

### PVAS

Coinciding with the reduction in on-animal and in-trap flea counts, there was a significant reduction in mean owner-reported pruritus scores after only 7 days following the first lotilaner treatment. In one home, a continued emergence of fleas in weeks 2 and 3 coincided with increased PVAS scores. Prior to any treatment, this home had 99 fleas counted in traps, and enrolled cats were scored 9.7 and 9.8 on the PVAS; 1 week post-treatment, trapped flea numbers dropped to 24 and the corresponding PVAS scores declined to 5.2 for both cats. Then, in weeks 2 and 3, flea-trap numbers rose to 68 and 89, respectively, and PVAS scores increased to 9.7 and 9.6 for one cat (respective flea counts of 2 and 0), and 6.2 and 5.3 for the other (flea counts 0 and 3). Trapped flea numbers then dropped to 1 and 0 in weeks 8 and 11–12, respectively, and scores for each cat were 0 on and after week 8. Given that the PVAS scores of cats with “high” and “low” flea burdens were not significantly different, the study findings highlight that a relatively low flea count, or perhaps more importantly, environmental exposure to fleas, can result in clinically evident pruritus in cats. The consistent use of PVAS scores in cats may be a useful way of monitoring changes in a home’s environmental flea burden.

This is the first study of which we are aware that included PVAS scoring of cats with no fleas. The validated PVAS score in “normal” dogs is ≤ 1.9, and in this study the mean PVAS score of cats from “no” homes was 1.9 (median 1.7) [23, 24]. This may be an appropriate baseline for monitoring the reduction of pruritus over time in cats with allergic dermatologic conditions. A limitation of this interpretation may be that only 14 cats were included in this group, 2 of which had PVAS scores of 5.1 and 4.3. Additionally, the study-end mean and median PVAS scores of cats in the “high” and “low” categories were much lower (respective means 0.5 and 0.8, medians 0.2 and 0.5). It may be that in the presence of fleas at enrollment, owners were sensitized to their cat’s pruritus. Then following elimination of the flea challenge, owners were impressed with the drastic reduction in pruritus, so their scoring declined from very high to very low. This might also explain why the final “high” and “low” category mean PVAS scores were significantly lower than the mean scores of the “no” category. Further study is needed to investigate our conclusions in this regard.

### SCORFAD

The SCORFAD scores from cats in “high” homes are consistent with those seen in previous publications looking at skin lesions associated with flea feeding [12, 13, 29]. The significant difference between cats in “high” and “low” homes prior to the first lotilaner treatment may speak to the volume and chronicity of the infestation, especially given initial PVAS scores that were not significantly different. If left untreated for another month, both the flea numbers and lesion severity of cats in “low” homes may have more closely matched the numbers and lesions in “high” homes that had already reached that point. The high group could also have an over-represented number of cats with true flea allergy, resulting in more significant lesions, although we did not test or select for this in the study. Furthermore, the Spearman’s correlation noted that there was no correlation between number of fleas on cats and the lesion severity. On the basis of prior experience of the investigators, this is likely due to the fastidious grooming behavior of cats. The grooming of fleas has been documented in laboratory studies, where cats readily remove free-roaming fleas, and cats with flea allergy remove more fleas than cats that are flea-naive [30, 31]. Given that cats in our study may have had significantly different initial flea burdens, there was no obvious correlation between flea counts and SCORFAD scores. Veterinarians should not rule out fleas as a cause of dermatologic lesions just because there are few, and possibly no, fleas seen during the exam. Flea control is an important diagnostic tool in working up dermatologic lesions. There was a consistent reduction in lesion severity over time in response to flea treatment, without any additional systemic or dermatologic treatments. Cats treated with lotilaner showed a significant reduction in lesion scores by the time of the first posttreatment assessment in week 4. The SCORFAD scores were less responsive to flea reduction than PVAS scoring because lesions, such as excoriations and self-induced alopecia, take time to fully resolve. In a clinical setting, lesions may improve faster if effective flea control is combined with other therapies such as antipruritic and immunosuppressive therapies. Regardless, the study-end SCORFAD scores of cats from “high” and “low” homes were not significantly different from the “no” home scores taken at enrollment. In the absence of a validated SCORFAD baseline for cats, the average SCORFAD score of 0.9 for the cats from “no” homes served as a comparative value for local cats and the dermatologic lesions seen during normal grooming behaviors. The reduction of the SCORFAD scores of flea-infested cats toward this presumed baseline is consistent with successful resolution of the lesions with only flea treatments and no other medical interventions. In practice, veterinarians have other drugs available to reduce inflammation and treat secondary infections associated with flea-feeding, but rapid and prolonged killing of fleas remains the key to stopping the progression of and reducing these dermatologic lesions.

## Limitations

Due to the nature of an in-home study, there are variables that cannot be controlled. Investigators must trust owners in regard to vacuuming and prior environmental and pet treatments. A limitation for “low” category homes is that there is not a standardized cutoff for the number of fleas that can be on an animal or a trap, and therefore may have a larger variation in dermatologic lesions. Furthermore, this investigation does not begin at a standardized time of the home flea infestation, thus, the duration of the infestation is unknown, which can produce variability in the environmental burden of fleas and severity of lesions. Despite this, there is a consistent, repeatable pattern of environmental flea control, pruritus reduction, and lesion resolution seen in this and previous studies using only highly effective flea control [12–18, 20].

## Conclusions

Over the 12-week study period, lotilaner treatment of infested cats and dogs was 100% effective at eliminating flea infestations from animals and their homes. This study illustrates the value of systematic flea control with lotilaner for up to 3 months to eliminate fleas from cats and to prevent reinfestation from the home environment. Flea-trap counts may be a more effective means of monitoring flea challenge to household cats than on-animal flea counts. Reductions in dermatologic and owner pruritis scores (PVAS and SCORFAD) demonstrated that the high lotilaner efficacy resulted in clinical resolution of flea-induced allergic dermatitis in cats.

## Data Availability

Data from this study are proprietary and are available on reasonable request to Elanco Animal Health.

## Abbreviations

FAD: Flea allergy dermatitis
PVAS: Pruritus visual analog scale
SCORFAD: Scoring feline allergic dermatitis
